# Quick Sepsis-related Organ Failure Assessment Versus Systemic Inflammatory Response Syndrome Criteria for Predicting Organ Dysfunction and Mortality

**DOI:** 10.7759/cureus.3511

**Published:** 2018-10-29

**Authors:** Punnavit Harimtepathip, James R Lee, Elliot Griffith, Gabriel Williams, Ravi V Patel, David Lebowitz, Sina Koochakzadeh

**Affiliations:** 1 Internal Medicine, University of Central Florida College of Medicine, Orlando, USA; 2 Emergency Medicine, University of Central Florida College of Medicine, Orlando, USA

**Keywords:** qsofa, sirs, predicting organ dysfunction, systemic inflammatory response, quick sequential organ failure assessment, sepsis

## Abstract

The International Consensus Definition for Sepsis and Septic Shock Task Force has recently developed new methods to determine whether a patient is at risk for end organ failure after he has been suspected to have sepsis. One of the newest measures developed is a quick Sequential (Sepsis-related) Organ Failure Assessment (qSOFA), and it is used to identify patients who are at risk of sepsis outside the intensive care unit.

The systemic inflammatory response syndrome (SIRS) score has previously been the standard for determining a patient’s sepsis risk and prognosis for future mortality. With the development of these new tools, it is imperative to compare qSOFA to SIRS in order to determine which method is best and under which circumstances. We conclude that according to evidence currently available, qSOFA has limited use for patients in the intensive care unit at the time of evaluation for predicting mortality and organ dysfunction. Furthermore, qSOFA outranks SIRS for patients in the emergency department except for SIRS delivering positive results more quickly.

## Introduction and background

In 2016, the European Society of Intensive Care Medicine and the Society of Critical Care Medicine (SCCM) created a task force that proposed Sepsis-3, a new definition for sepsis [1]. Now defined as a life-threatening organ dysfunction caused by dysregulated host response to infection, Sepsis-3 no longer involves the use of systemic inflammatory response syndrome (SIRS) criteria. SIRS was originally used in the 1991 definition of Sepsis-1 in which the criteria included tachycardia (heart rate (HR) > 90 beat/min), tachypnea (relative risk (RR) > 20 breaths/min), fever (temp. <36 or >38℃), and leukocytosis or leukopenia (white blood cell count (WBC) > 1,200/mm^3^ or <4000/mm^3^). Patients meeting two or more of these criteria along with infection or suspected infection met Sepsis-1 criteria. Sepsis-2 was introduced in 2002, and it included expanded diagnostic criteria but was otherwise the same as Sepsis-1. The 2016 task force compared SIRS to sequential organ failure assessment (SOFA) and logistic organ dysfunction system (LODS). SOFA was recommended for assessing organ dysfunction severity. The task force also claimed that the predictive validity of SOFA is superior to SIRS criteria among other advantages of this diagnostic tool [2]. Some studies have shown that SOFA is superior in terms of predicting in-hospital mortality and greater prognostic accuracy for in-hospital mortality than SIRS criteria [2-4]. Due to the complexity of SOFA, a lack of data for diagnostic criteria for many patients, and the possibility of delay in identification of sepsis, the task force recommended use of “quick SOFA” or qSOFA.

There are controversies regarding the superiority of SIRS versus qSOFA. Studies have shown that patients meeting SIRS criteria had an increased risk of organ dysfunction and mortality in those without known organ dysfunction while qSOFA was poorly sensitive for organ dysfunction [5]. Other studies have shown qSOFA may be inferior in terms of early identification of sepsis [6]. The recently published Sepsis-3 criteria along with many criteria and scoring systems for the evaluation of patients with suspected sepsis demonstrate the need for a consensus on the hierarchy of the most accurate and efficient prognostic criteria and/or scoring systems has become apparent. This paper seeks to examine how qSOFA and SIRS criteria compare in their ability to predict organ dysfunction and/or failure based on evidence-based literature.

## Review

Mixed results for qSOFA versus SIRS

Due to the need for a protocol in choosing the correct evaluation system for patients with sepsis-like symptoms, Raith et al. sought to assess the discriminatory capacity of SOFA, qSOFA and SIRS criteria in patients who were suspected to have an infection and were observed to have an increase by two or more criteria/points within the first 24 hours of admission [7]. The primary outcome measured was in-hospital mortality. This was a retrospective cohort study consisting of 184,875 adults at least 17 years of age who were admitted to the intensive care units at 182 Australian and New Zealand intensive care units with suspected infection. The data used was gathered from the Australian and New Zealand Intensive Care Society (ANZICS) Adult Patient Database and included records from 2000 to 2015. SOFA, qSOFA and SIRS criteria were calculated using lab and physiological parameters recorded from the first 24 hours of intensive care unit (ICU) admission. Patients that were repeat ICU admissions and those that were transferred were excluded from the study [7].

The primary outcome was in-hospital mortality, while the (composite) secondary outcome was ICU length of stay of three days or more. Discriminatory power of each scoring system was determined by comparing the area under the receiver operating characteristic curve (AUROC) for each individual score (unadjusted analysis) together with a baseline risk model (adjusted analysis).

The discrimination of in-hospital mortality for SOFA (75.3% AUROC; 99% confidence interval (CI): 0.750–0.757) was significantly higher than that of qSOFA (60.7% AUROC; 99% CI: 0.603–0.611) or SIRS (58.9% AUROC; 99% CI: 0.585–0.593). Of the study population, 90.1% (165,103 patients) had an increase in SOFA score from baseline to at least two points; 86.7% (158,710 patients) met two or more SIRS criteria, and 54.4% (99,611 patients) had a qSOFA score of at least two points [7]. In adults admitted to the ICU with suspected infection, an increase in SOFA score of at least two points had superior prognostic accuracy for in-hospital mortality followed by qSOFA and finally SIRS criteria. With SOFA score demonstrating significantly greater discrimination for in-hospital mortality, the authors highlight that this may suggest that SIRS criteria and qSOFA may have limited utility in predicting mortality in an ICU setting [7].

Some of the strengths of this study was the large sample size, with a sample size of 184,875 patients admitted with suspected infection over approximately 16 years. In addition, the use of clinical diagnoses rather than coding data in identifying patients served to increase the study’s external validity and applicability. One limitation of this study was that the data analyzed was that from within 24 hours of admission and thus, the accuracy of the link between physiological metrics and scoring/criteria met could be diminished. Another limitation is that the SOFA score used in this study was modified because the cardiovascular components were estimated due to unknown inotrope or vasopressor doses administered.

Although this study focuses primarily on patients admitted to the ICU with suspected infections, its impact is significant due in part to the fact that infection-related illnesses are among the most prevalent and are a common cause for hospital and ICU admissions worldwide. The stratification of discriminatory prognostic ability of SOFA, qSOFA and SIRS criteria has the potential to be a useful tool in ICUs and will lead to more efficient and accurate recognition of patients in danger of critical organ failure and death.

April et al. compared qSOFA versus SIRS using AUROC, sensitivity, specificity and likelihood ratios to determine which was most effective in predicting in-hospital mortality in emergency department (ED) patients with suspected infection that were admitted to the ICU. The study was a retrospective cohort chart review and the chart abstractors were blinded to the study hypothesis to rid the study of bias. The review included 214 patients that were admitted to the ICU with presumed sepsis from August 2012 to February 2015. Patients were included if they had a body fluid culture sampled during their ED stay with or without intravenous (IV) antibiotics. If they were administered antibiotics, the culture had to have been taken within 24 hours of the antibiotics administration. The primary outcome of the review was to get prognostic accuracy of qSOFA and SIRS for predicting in-hospital mortality. Secondary outcome included assessment of prognostic accuracy of LODS and SOFA, two different prognostic tools [8].

The researchers obtained all data by chart review from the San Antonio Military Medical Center. The study population was composed of ED patients admitted to an ICU setting. The researchers concluded that SIRS and qSOFA were comparable in prognostic value for predicting in-hospital mortality. They essentially are about the same in sensitivity and specificity according to this study. The sensitivity for two SIRS criteria was 97.4% and 89.7% for two qSOFA criteria. Both had low specificities at 2.5% and 27.4%, respectively. The prognostic superiority of qSOFA over SIRS does not appear to hold when applied to the sickest ED patients requiring ICU care [8].

This is important for ED physicians since they deal with the whole population prior to admission to ICU or wards so they need to know that qSOFA may cause them to miss some critically ill patients. Benefit of qSOFA is lack of need for lab values and better prognostic value in ED patient that are not admitted to ICU. This external validation study of SIRS and qSOFA in ED patients admitted to an ICU finds similar prognostic accuracy for in-hospital mortality as reported by the Sepsis-3 guidelines for ICU patients. The SIRS criteria appear non-inferior to the qSOFA criteria in this patient population.

Churpek et al. did an observational cohort study that sought to compare qSOFA with other used early warning scores. They compared qSOFA, SIRS, modified early warning score (MEWS) and national early warning score (NEWS) in predicting prognosis and ICU transfer. Patients were eligible for inclusion in the study if they were admitted to the University of Chicago urban tertiary care medical center between November 2008 and January 2016 and if they were suspected for sepsis some time during their stay. Patients without lab or vital signs documented in the ED or wards were excluded from the study. Patients who had also received mechanical ventilation or vasopressor medications before suspicion of infection were excluded because a prognostic tool would not offer additional value for those patients because they would have been admitted directly to the ICU [6].

In the study, 30,677 patients were included and the primary outcome for this study was in-hospital mortality, and the secondary outcome included death of the patient or an ICU stay at any point after meeting suspicion for infection criteria. The effectiveness of each detection method to detect in-hospital mortality and ICU transfer was as follows (sensitivity & specificity): NEWS (67% & 66%), MEWS (59% & 70%), qSOFA (54% & 67%), and SIRS (91% & 13%) [6].

The authors concluded qSOFA should not replace NEWS or MEWS (general early warning scores) for risk-stratifying patients with suspected infection [6]. The fact that SIRS is non-specific, with 90% of ICU patients and 50% of all ward patients fitting the criteria, this was also not a satisfactory prognostic tool due to overestimation of adverse outcomes in patients without risk for organ dysfunction. The advantage that qSOFA has over MEWS/NEWS was the simplicity of the criteria and reported errors in using the MEWS/NEWS calculations. Limitations to the study include the fact that it is a single-center investigation. Also, since there is no gold standard to determine patient infection, it is possible that candidates were excluded from the study when they should have been included. The main strength of this study was the sample size [6].

In a study of consecutive ED patients who were admitted with presumed infections, primary outcomes of 30 days and one year mortality were measured in a prospective study with 8,871 patients [5]. Williams et al. sought to determine the prognostic value of SIRS, qSOFA, SOFA. SIRS was established in 47.1% of patients. SIRS showed relative risk (RR) of 3.5 for increased risk of organ dysfunction and an odds ratio of 3.2 for predicting mortality in patients without organ dysfunction. For the purpose of our evaluation, the SIRS criteria versus qSOFA criteria was highlighted and analyzed based on their ability to predict end organ failure. In these researchers’ evaluation, SIRS and qSOFA showed that they both were able to discriminate well for organ dysfunction (AUROC .72 and .73). However, qSOFA was specific, but not sensitive for organ dysfunction with 96.1% specificity and 29.7% sensitivity. This study made recommendations that the removal of SIRS as a screening tool for organ dysfunction and death in the ED was not warranted. A qSOFA score greater than or equal to two had low sensitivity which undermines its use as a bedside tool in the ED. This study was limited by being a single site study although the sample size was sufficiently large [5].

Haydar et al. analyzed health records from 200 patients treated in the ED. Two hundred patients were randomly selected from a population of 1,880 patients, who were ultimately discharged from the hospital with a diagnosis of sepsis. In their analysis they found that it takes up to 37 minutes longer for a patient in the ED to meet qSOFA criteria than to meet SIRS criteria [9]. The qSOFA is a good tool to identify those with sepsis that have a higher risk of mortality however it does not seem to be the best screening tool for early identification of sepsis in the ED. If any of the qSOFA measures are present then it most often means there is a severity of illness that usually is not in the earliest phases of the disease process. Relying on qSOFA may delay diagnosis and initiation of interventions. Evidence shows that early intervention with antibiotics and fluids improves patient outcomes in sepsis. A delay in these interventions results in worsened outcomes in patients [9].

In support of qSOFA over SIRS criteria

Freund et al. conducted a prospective study of the use of qSOFA as a predictor of in-hospital mortality, in the emergency department specifically, compared to SIRS and the previous severe sepsis criteria [3]. A total of 879 patients from 30 emergency departments were included in this international study to assess qSOFA validity as a screening tool for end-organ failure. In that timeline, consecutive patients with suspected infection were included in this study and assessed based on the qSOFA, SOFA, and SIRS criteria. The three components of qSOFA (lowest blood pressure < 100, highest respiratory > 21, and lowest Glasgow Coma Score that’s <15) were collected. The primary end-point measured was in-hospital mortality. Secondary endpoints included admission to ICU, length of ICU stay of more than 72 hours, and a composite death or ICU stay of more than 72 hours. All patients were followed up until hospital discharge or death. The aim of this study was to validate the results of the Sepsis-3 consensus article hypothesizing patients with a qSOFA score of two or higher who have an in-hospital mortality rate of at least 10%.

Of 1,088 patients screened, 879 were included in the analysis. Overall in-hospital mortality was 8%: 3% for patients with a qSOFA score lower than two versus 24% for those with qSOFA score of two or higher (absolute difference, 21%; 95% CI: 15%–26%). The qSOFA performed better than both SIRS and severe sepsis in predicting in-hospital mortality, with an area under the receiver operating curve (AUROC) of 0.80 (95% CI: 0.74–0.85) versus 0.65 (95% CI: 0.59–0.70) for both SIRS and severe sepsis (P < .001; incremental AUROC, 0.15; 95% CI: 0.09–0.22). The hazard ratio of qSOFA score for death was 6.2 (95% CI: 3.8–10.3) versus 3.5 (95% CI: 2.2–5.5) for severe sepsis [3].

This report concluded that the prognostic accuracy of qSOFA for mortality in patients presenting in the ED was greater than that of SIRS and severe sepsis [3]. These findings support the consensus provided by the Sepsis-3 criteria in the emergency department. The limitations of this study include the lack of follow-up of discharged patients as the study only tracked in-hospital mortality. Also, only the worst qSOFA value during the ED stay was used for the study, this made it impossible to track qSOFA’s ability to detect sepsis in a timely manner. Further studies have been done to ascertain the timeliness of qSOFA as a screening tool.

Seymour et al. sought to validate current clinical criteria for identifying patients with suspected infection and are at risk for sepsis. The researchers looked at electronic health records (EHRs) and assessed SIRS, qSOFA and other detection methods (LODS and SOFA). They concluded that in ICU patients with suspected infection that SOFA and LODS were similar in rank but both outdid SIRS and qSOFA for predicting in-hospital mortality. However, for patients with suspected infection outside the ICU the predictive validity for in-hospital mortality of qSOFA was greater than SOFA and SIRS. The Third International Consensus definition task force recommended use of SOFA score two or more in encounters in the ICU to help predict sepsis and then use of qSOFA in non-ICU setting to consider the possibility of sepsis [10].

SOFA and LODS require lab and clinical data that may be hard to obtain in a timely manner for those in the ED. qSOFA is simple and only uses three clinical variables with no lab tests and does better at predicting sepsis outside of the ICU. qSOFA is not intended to determine whether someone has an infection or not. It is meant to be used in those who have a suspected infection. Luckily, suspicion of infection is high in hospitals internationally which explains the rapid initiation of antibiotics in many institutions. Furthermore, qSOFA gives a point for anyone with abnormal mental status (Glasgow coma score (GCS) less than or equal to 13) and does not take into account baseline status. This makes that aspect of the test hard to validate. Reasons for qSOFA not being useful in the ICU setting are because it takes into account less variables and no lab data as compared to LODS and SOFA. Also, there are the confounding effects of ICU patients being on vasopressors or having mechanical ventilation which makes the qSOFA clinical variables somewhat tainted [10].

Moving forward with the advances in EHRs, hospitals can now design functions in the health records to use these tools (qSOFA, SOFA, SIRS) to flag patients that may be at risk and to alert physicians that patients with infections may be entering into sepsis. In addition, this article mentions that the addition of serum lactate levels helps improve the predictive validity of qSOFA. This additional metric could help distinguish patients with borderline qSOFA scores.

The retrospective observational study by Park et al. determined the prognostic value of qSOFA compared with SIRS as it related to organ failure in patients with suspected infection in the ED. Over 1,000 patients were included in the study and it found that the validity of qSOFA (AUROC = .814) was higher than SIRS (AURUC = .662). As opposed to other studies, qSOFA had respectable sensitivity of 75% and specificity of 82% for end organ failure [10]. They concluded that qSOFA was superior to SIRS in predicting organ failure in ED patients with suspected infection. They found that qSOFA also was better at predicting in-hospital mortality than SIRS. Park et al. recommend a cutoff value for qSOFA equal to or greater than one to predict organ failure for patients with a suspected infection. However, qSOFA has a low sensitivity and negative predictive value (NPV) which means there must be further confirmatory tests to determine organ failure [10].

Finkelsztein et al. compared qSOFA and SIRS for their ability to predict mortality, ICU free days and organ dysfunction free days in patients with a suspected infection outside of the ICU. The patient group was from Cornell’s biobank of critically ill patients but the calculations were done prospectively on patients in the ED or hospital wards who were suspected to have infection and were admitted to the ICU [4]. There was no blinding in this study. This study was funded by a National Institutes of Health grant. A total of 152 patients from the Weill Cornell Medicine Registry and Biobank of Critically Ill Patients registry were included in this study. Inclusion criteria included only patients eligible for the registry that were suspected of having an infection. This includes adults greater than 18 years old admitted to the ICU and willing to provide biological samples for purpose of research. Exclusion criteria from the registry include those who could not provide informed consent, cognitively impaired, unwilling to receive blood transfusion, or hemoglobin less than 7 g/dL. Furthermore, for this current study, those transferred from an outside hospital or those coming from the OR were excluded from this study. The primary outcome of this study was all-cause in-hospital mortality. Secondary outcomes included ICU-free days from ICU admission to day 28, ventilator-free days, organ dysfunction-free days, and renal dysfunction-free days [4].

Discrimination of in-hospital mortality for qSOFA was AUC of 0.74 with 95% CI of 0.66–0.81 and for SIRS was AUC of 0.59; 95% CI of 0.51–0.67. For ICU-free days, discrimination of qSOFA (AUC of 0.65 with 95% CI: 0.57–0.72) was greater than SIRS criteria (AUC, 0.54; 95% CI: 0.45–0.62; p = 0.04). The ventilator-free day discrimination was not different for qSOFA (AUC, 0.64; 95% CI: 0.56–0.71) versus SIRS (AUC of 0.57, 95% CI: 0.48–0.65). There was also no difference for any organ dysfunction-free days between qSOFA (AUC of 0.59; 95% CI: 0.51–0.67) and SIRS criteria (AUC, 0.51; 95% CI: 0.42–0.59; p = 0.13).

The researchers concluded that qSOFA outperformed SIRS in the prediction of in-hospital mortality and ICU-free days. However, qSOFA was not reliable in predicting ventilator-free days, organ dysfunction-free days or renal dysfunction-free days. SIRS is too sensitive and it identifies people with normal regulatory responses that are not in sepsis. The limitations of this study include the use of all-cause mortality instead of sepsis-related mortality and patients suspected of having sepsis instead of all critically ill patients for study outcome and population [4]. Also, qSOFA and SIRS score could not be calculated before eight hours prior to ICU admission. Additionally, qSOFA and SIRS scores are not mutually exclusive and about 61% of patients in this study met both criteria. ICU transfers could not serve as an outcome because all of the patients included were eventually admitted to the ICU.

## Conclusions

This review discussed results of several studies that evaluated the effectiveness of qSOFA in comparison to other methods of determining sepsis patient prognosis. Based on this review, the SIRS criteria are more sensitive across both the ICU and in hospital setting while qSOFA is more specific across both settings and a better predictor of mortality. qSOFA outperforms SIRS when it comes to predicting mortality, although it lacks superiority in predicting mortality in the sickest ED patients requiring ICU care. Despite its low sensitivity, qSOFA score is still a useful tool and has prognostic value in patients outside the ICU, ED, as well as patients who would eventually end up in the ICU. A disadvantage of qSOFA is a possible delay in the diagnosis of sepsis which may lead to more adverse health outcomes. Both qSOFA and SIRS criteria lacked efficacy when used in sick ICU patients. There is a need for further studies to help establish a clear consensus or a new and more efficacious criteria; however, qSOFA, at the very least, can be used by physicians, especially in ED, to help guide their assessment of a patient's risk for in-hospital mortality.
